# Global challenges and microbial biofilms: Identification of priority questions in biofilm research, innovation and policy

**DOI:** 10.1016/j.bioflm.2024.100210

**Published:** 2024-07-04

**Authors:** Tom Coenye, Merja Ahonen, Skip Anderson, Miguel Cámara, Parvathi Chundi, Matthew Fields, Ines Foidl, Etienne Z. Gnimpieba, Kristen Griffin, Jamie Hinks, Anup R. Loka, Carol Lushbough, Cait MacPhee, Natasha Nater, Rasmita Raval, Jo Slater-Jefferies, Pauline Teo, Sandra Wilks, Maria Yung, Biofilm Priority Questions Exercise Participants, Jeremy S. Webb

**Affiliations:** a Laboratory of Pharmaceutical Microbiology, Ghent University, Belgium; b ESCMID Study Group on Biofilms (ESGB), Basel, Switzerland; c Satakunta University of Applied Sciences, Finland; d Center for Biofilm Engineering, Montana State University, Bozeman, MT, USA; e University of Nebraska, Omaha, NE, USA; f National Biofilms Innovation Centre, School of Physics and Astronomy, University of Edinburgh, Edinburgh, UK; g National Biofilms Innovation Centre, University of Nottingham Biodiscovery Institute, School of Life Sciences, University of Nottingham, Nottingham, UK; h University of South Dakota, Vermillion, SD, USA; i Nanyang Technological University, Singapore; j Singapore Centre for Environmental Life Sciences Engineering (SCELSE), Singapore; k National Biofilms Innovation Centre, School of Biological Sciences, University of Southampton, Southampton, UK; l National Biofilms Innovation Centre, Open Innovation Hub for Antimicrobial Surfaces, Department of Chemistry, University of Liverpool, Liverpool, UK

## Abstract

Priority question exercises are increasingly used to frame and set future research, innovation and development agendas. They can provide an important bridge between the discoveries, data and outputs generated by researchers, and the information required by policy makers and funders. Microbial biofilms present huge scientific, societal and economic opportunities and challenges. In order to identify key priorities that will help to advance the field, here we review questions from a pool submitted by the international biofilm research community and from practitioners working across industry, the environment and medicine. To avoid bias we used computational approaches to group questions and manage a voting and selection process. The outcome of the exercise is a set of 78 unique questions, categorized in six themes: (i) Biofilm control, disruption, prevention, management, treatment (13 questions); (ii) Resistance, persistence, tolerance, role of aggregation, immune interaction, relevance to infection (10 questions); (iii) Model systems, standards, regulatory, policy education, interdisciplinary approaches (15 questions); (iv) Polymicrobial, interactions, ecology, microbiome, phage (13 questions); (v) Clinical focus, chronic infection, detection, diagnostics (13 questions); and (vi) Matrix, lipids, capsule, metabolism, development, physiology, ecology, evolution environment, microbiome, community engineering (14 questions). The questions presented are intended to highlight opportunities, stimulate discussion and provide focus for researchers, funders and policy makers, informing future research, innovation and development strategy for biofilms and microbial communities.

## 1 Introduction

Biofilms are structured communities of microbial cells, embedded within a self-produced matrix of extracellular polymers and other biomolecules. They are complex and dynamic, have a physical three-dimensional and aggregated architecture, and may contain many species and genotypes evolving and interacting with each other and with their environment [[1], [2], [3]]. Biofilms are the prevalent form of microbial life on Earth and drive the dynamics, activity and function of microbial communities and microbiomes underpinning all ecosystems [4]. They impact on major global challenges including climate change, safe and secure water and food, human and animal health, and antimicrobial resistance (AMR) [[5], [6], [7], [8]]. As such the potential scientific and societal benefits that could result from understanding, controlling or harnessing the activities of complex biofilms are considerable. Recent years have seen an unprecedented increase in our ability to describe and understand biofilms and their interactions with their environment or host. This has been made possible through technical advances for example in high-throughput, data-driven ‘omics’ approaches, high-resolution imaging and advanced spectroscopy to characterize their structure, activity, function and emergent properties. The intersection of biological mechanisms and functions of biofilms, together with their protective physical structure and presence, also creates unique problems and opportunities for biofilms and has led to the recognition that highly interdisciplinary approaches and teams are required to address many of these challenges. Huge opportunities remain, and the potential for new research avenues as well as for new beneficial impacts across diverse sectors is vast [9,10].

The purpose of this priority question exercise was to assist in identifying future important questions and avenues that biofilm studies could and should be addressing and that will have the greatest impact in advancing the field. Our approach was inspired by previous initiatives, which have used specific criteria to identify priority research questions to advance the field of a given discipline [[11], [12], [13]]. However, to date, there has not been an international community-wide synthesis of key questions and priority research or innovation areas for the biofilm field. In our call for questions we asked for questions (less than 100 words) that are currently unanswered within the scientific literature, could be answered (including through high-risk and blue skies research), and that could reasonably be tackled by a research programme. The stated goals of the exercise were to 1) stimulate discussion amongst the biofilm community and identify areas of research and innovation that would have a substantial scientific and societal impact; 2) to encourage researchers to think beyond the limits of their own sphere of research or discipline and consider the most important basic or applied research that could be carried out, and 3) To illustrate the most impactful and beneficial research in the field and its overall importance to funding agencies, policy makers, regulators and the wider public. Our definition of microbial biofilms was as inclusive as possible and included communities of bacteria that may be surface or interface-associated or suspended as aggregates, comprising single-species or polymicrobial consortia, and relevant to any fundamental or applied context in which biofilms are studied. By assembling this set of questions, we aim to stimulate discussion amongst members of the biofilm community and identify areas of research and innovation that are likely to have a substantial scientific impact. We also hope that these priority questions highlight the potential wider impact of biofilm research and its importance to funding agencies, policy makers, regulators and the wider public.

## 2 Methods

### 2.1 Background

At a joint meeting of biofilm centers held in Arlington, VA, USA in February 2020, representatives from the National Biofilms Innovation Centre (NBIC, UK), the Centre for Biofilm Engineering (CBE, USA) and the Singapore Centre for Environmental Life Sciences Engineering (SCELSE, Singapore) decided to coordinate a priority questions exercise to be published as a position paper and resource for the field. A core working group was established in the Fall of 2020, consisting of representatives of these biofilm centers, representatives from the ESCMID Study Group on Biofilms (ESGB) and the AmiCI COST Action, and several text modelers and data scientists. This core working group supervised the entire exercise, from developing the approach outlined below to the writing of this final report. At every step, the core working group aimed at taking into account geographical diversity, diversity in terms of field, disciplines and vocational backgrounds (with the aim to give opportunity to academics, practitioners, industry stakeholders, relevant organizations and policy makers to be included), and gender diversity. It was decided that there would be no limit to the number of questions that could be included on the list.

### 2.2 Set up of the submission form and invitation of participants

**Fig. 1:**
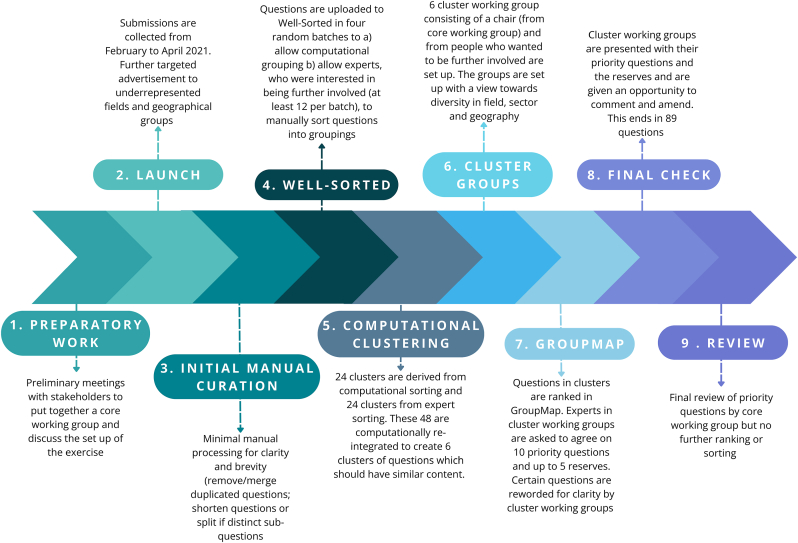
Biofilm priority question exercise process flow diagram.

For the submission form, different options were considered but taking into account applicable data protection rules, MS Forms was chosen. The form was set up to allow participants to add multiple entries (up to three at one time and if participants wanted to submit more entries, they could redo the questionnaire). At the time of submitting their entries, participants were given the opportunity (in a separate form, so entries could be kept anonymous) to get involved in further steps. A copy of the empty submission form is shown in Supplementary Fig. S1. Participants were invited to contribute by announcements across several social media platforms (for example, Supplementary Fig. S2) and the websites of the participating biofilm centers, and by invitations sent out to mailing lists of the participating entities. In addition, members of the core working group sent out invitations through their own network. An example of an invitation email is shown in Supplementary Fig. S3. Furthermore, to provide additional context and an overview of the question section process, we have also included a process flow diagram (Fig. 1). Questions were collected from February to April 2021.

### 2.3 Sorting of submissions

Prior to the actual sorting, duplicate questions were removed/merged and some questions were shortened (i.e. narrowed down to the actual question and/or split in two or more separate questions if appropriate); this was done by members of the core working group. To avoid human bias as much as possible, the resulting set of questions was sorted using ‘Well Sorted’ (https://www.well-sorted.org/index.php). To this end, the questions were split randomly into four batches (designated A, B, C and D) and for each batch, 12 experts (members of the core working group as well as participants who had indicated they wanted to be further involved) were asked to group the questions within a batch into different themes. During this process, each participant was given the freedom to determine the number of themes within their set of questions. The questions and sorting results were then re-integrated computationally, leading to 24 groups of questions. In addition, an average linkage algorithm was employed to integrate the four batches of questions (A, B, C, D), leading to an additional set of 24 groups generated using a computational approach only. These 48 groups were subsequently merged to create six clusters via a computational process. In a final step, members of the core working group performed a final manual curation step. In this final step a few remaining duplicate questions (not identified before) were removed, and several questions were moved from one group to another, to increase internal coherence within the six groups. Details about the computational procedure will be published separately.

## 3 Results

We received 173 submissions. As people were able to submit multiple questions in one entry, this meant that 279 questions were submitted. Questions were received from 31 countries (Argentina, Australia, Belgium, Brazil, Canada, China, Czech Republic, Denmark, Ecuador, Estonia, France, Germany, India, Israel, Italy, Lithuania, Mexico, Netherlands, New Zealand, Pakistan, Portugal, Saudi Arabia, Serbia, Singapore, Slovakia, South Africa, Spain, Sweden, Ukraine, UK, USA) representing every continent. The 279 initial questions were in a first step split into 309 questions (as several of the 279 original questions contained distinct sub-questions). The process described above ultimately led to a collection of 78 unique questions that were grouped in six thematic categories, each containing between 10 and 16 questions. Each category, as well as each question within a category, received a number, but neither the categories nor the questions are ranked in any way (e.g. Q1.1 is not by definition more important or relevant than Q5.10). The grouping in thematic categories should be seen as an attempt to cluster questions addressing the same topic(s), but we are aware that alternative groupings could be equally meaningful. For some questions consisting of several related sub-questions, it was decided to keep these together, rather than artificially putting them in separate questions or categories (e.g. Q2.2).

### 3.1 Biofilm control, disruption, prevention, management, treatment

The questions in this category reflect that an improved ability to control and harness biofilms will impact on some of the most important global challenges, including climate change, safe and secure water and food supplies, and positive and negative aspects in relation to human and animal health. For example, microorganisms represent >20 % (93.2 Gt carbon) of the total global biomass on Earth [14] of which approx. 80 % exists as biofilms [4] and which dominate biogeochemical cycling. Therefore, in the context of global targets to reduce greenhouse gas emissions to net-zero, coupled with commitments to protect and enhance the environment, and rebuild biodiversity (for examples see Refs. [[15], [16], [17]]), the biofilm field has a unique opportunity to play a leadership role in protecting and improving the health of our environment. Academic-industry partnerships and know-how provide the opportunity to address these challenges in a multi- and interdisciplinary manner and at scale. One example is the potential role of soil microbiology in promoting both the health and productivity of soil and its ability to store carbon [18,19]. Other areas where new understanding and control of microbial biofilm processes would have a major impact include microbially influenced corrosion. This creates huge economic problems through failure of marine and energy sector infrastructure [10] but microbially influenced corrosion remains challenging to predict, detect or prevent [20,21]. The potential for biocatalytic applications of biofilms is also raised, and it is clear biofilms provide promising alternatives strategies in the production of biofuels and value-added compounds [22,23]. Finally, there is huge potential for research, development and innovation using new and transformative technologies (e.g. novel nanotechnology, CRISPR-based gene editing approaches) that may provide a step change in our ability to modulate or control biofilm development and life cycle dynamics.

The identified priority questions in this category are:

1.1 How can biofilms and microbial communities be exploited to address climate change challenges?
1.2 Planktonic culturing has long been used in biotechnology for production of valuable biologics. Can biofilms be used for this as well?
1.3 How can we exploit the latest understanding of biofilms and microbiomes for next generation agricultural practices and integrate them with the regenerative farming practices?
1.4 Can detailed knowledge of biofilm genetics, physiology, ecology etc. be applied to develop *in situ* bioreactors for bioremediation that have extended sustainability, enhanced performance, and minimal site impact?
1.5 Is disrupting biofilms good enough as a therapeutic treatment or do we need biofilm killing?
1.6 Could we use nanomaterial-based drug-delivery systems to treat biofilm-associated infections?
1.7 Can we identify new concepts to prevent biofilm formation (e.g. new materials/surfaces/coatings to disrupt biofilm life-cycle dynamics)?
1.8 What could CRISPR provide in terms of opportunities for the removal/eradication of biofilms?
1.9 Can benign/commensal bacteria be used to invade and clear pathogenic biofilms?
1.10 How can we predict and control microbially influenced corrosion?
1.11 What is the abundance and community composition of pathogenic bacteria and viruses on microplastics collected from marine environments?
1.12 How widespread are ‘dry biofilms’, that are being increasingly reported on hospital surfaces? Are these found in other built environments (e.g. food processing, transportation) or in nature (e.g. in extreme environments such as those found in Antarctica)?
1.13 How do biofilms formed under conditions of microgravity (e.g. in space) differ from those formed under Earth gravitational conditions, and what consequences do these potential differences have on pathogenic processes and material degradation?

### 3.2 Resistance, persistence, tolerance, role of aggregation, immune interaction, relevance to infection

An improved understanding of the interaction of the host immune system with microbial biofilms is crucial for better understanding the mechanisms behind chronic infections [[24], [25], [26]] as well as for the development of novel therapeutic and preventive approaches, including vaccines [27]. Reduced susceptibility to antimicrobial agents (including antibiotics and disinfectants) is a hallmark of biofilm-related infections [5,[28], [29], [30]] and is due to a combination of resistance, tolerance, and persistence [[30], [31], [32], [33]]; however, the exact contribution of these processes is not always clear. In addition, while increased horizontal gene transfer has been observed in biofilms, it is currently unclear how this contributes to the overall spread of antimicrobial resistance genes [30]. Guidelines for improved diagnosis of biofilm-associated infections have been published [34,35], but it remains to be determined whether they will play a major role in clinical decision making. Likewise, biofilm antimicrobial susceptibility testing is receiving increasing attention, but in the absence of standardized approaches and clinical breakpoints, its implementation in clinical practice remains difficult [[36], [37], [38], [39]]. A lot of biofilm research is focused on biofilm elimination, both in clinical and environmental settings, but this remains difficult to achieve. Further research into various forms of combination therapy, as well as input from a wide range of research disciplines will be needed to make progress in this area. Finally, over the past decades convincing evidence has been presented confirming the involvement of microbial biofilms in many (chronic) infections [40,41]. However, we know that biofilms can take many forms (e.g. biotic or abiotic surface attached, suspended aggregates) [1] and it remains to be determined how similar the mechanisms underlying the formation of these different types of biofilms are.

The identified priority questions in this category are:

2.1. How can we make organisms in biofilm infections accessible to the immune system?
2.2. How does the immune response differ between planktonic and biofilm-related infections? Are antibodies being produced against biofilm matrix compounds?
2.3. What are the *in vivo* and *in vitro* tools that could help elucidate the mechanisms behind immune evasion for the different pathogens involved in biofilm-related infections? How can we use this knowledge to improve current treatment approaches?
2.4. Microbial cells in biofilms and tolerant persisters share many common properties. What is the link between persisters and biofilms?
2.5. What is the contribution of microbial biofilms to the development, maintenance and spread of antimicrobial resistance and tolerance?
2.6. How can confirmation of the presence of a biofilm help clinicians to make treatment decisions?
2.7. Is it possible to introduce biofilm antimicrobial susceptibility testing and define specific clinical breakpoints in routine diagnostics?
2.8. What is the added value of combining technologies (e.g. chemical and mechanical action) for a more environmentally friendly solution to biofilm elimination?
2.9. How do we more effectively combine various research disciplines (e.g. biological and physical sciences, engineering, pharmaceutical sciences, medicine etc.) to further develop antibiofilm research and to enhance translation of existing knowledge into clinical practice?
2.10. Do biofilms form by similar mechanisms at different sites (e.g., wound exudates, cystic fibrosis sputum, vascular or urinary catheter, tooth surfaces) or are these fundamentally different processes?

### 3.3 Model systems, standards, regulatory issues, policy education, interdisciplinary approaches

Many model systems are available to study biofilms under laboratory conditions, ranging from simple (e.g. microtiter plate based models) to more complex (e.g. animal models) systems [[42], [43], [44], [45]]. In addition various (clinical) conditions have been mimicked by a range of different models, for example in the context of biofilm-related wound infection [[46], [47], [48]], biofilms in root canals [49], and caries-related biofilms [50]. The accuracy (i.e. the *in vivo*-likeness) of any given model system can be assessed using quantitative frameworks based on RNA sequencing data and these frameworks are starting to be developed [51]. Standardization of biofilm methods is important, especially in (but not limited to) studies addressing anti-biofilm activity of certain approaches or compounds [[52], [53], [54], [55], [56]] and in combination with continuous interactions with regulatory agencies, standardization will be key to the further translation of fundamental biofilm research into applications [57,58]). A recent (2019) analysis estimated that the economic significance of biofilms is in excess of $5000bn a year [10] and the annual (2010) incidence of and mortality due to biofilm-associated diseases in the US alone was estimated to be 17 million and 550.000, respectively [41]; yet the feeling among many in the biofilm field is that this enormous impact is still not recognized sufficiently. This may be partially due to the biofilm community being ‘*too scattered*’ and ‘*lacking the unity found in other research topics*’ [9]. The further promotion of the biofilm concept by centers and consortia like NBIC, CBE, SCELSE and ESGB [58,59] as well as educational outreach activities [60] will be essential to increase the awareness of the considerable impact biofilms have. Essential in this context is the use of up-to-date definitions and conceptual models [1].

The identified priority questions in this category are:

3.1 How can we more accurately model biofilms *in vitro*, in order to better reflect the *in vivo*/real world situation?
3.2 Can there be a unified definition of biofilms, encompassing surface-attached biofilms, suspended aggregates etc.? Or should we have multiple definitions? What should that definition (or those definitions) be?
3.3 How much do the differences between the biofilm vs planktonic lifestyle (and the different methods used to study them) affect current antimicrobial testing and research in general?
3.4 How do we define which *in vitro* biofilm behaviors are important in the *in vivo* biofilm context, to further dissect these behaviors?
3.5 How do different growth methods (e.g., multi-well plates with different volume to surface ratio, flasks vs test tubes, resuspension etc.) impact biofilm characteristics?
3.6 How can standardized method(s) be developed for anti-biofilm susceptibility testing and what evidence is needed to substantiate claims about cleaning, removal, eradication, clinical efficacy etc. To regulatory agencies and other stakeholders?
3.7 How can we get clarification and increased awareness of standards and regulatory pathways for biofilms from industry regulators?
3.8 Are current testing methods for antimicrobial materials fit for purpose with respect to anti-adhesion, anti-biofilm claims?
3.9 How can we detect and confirm the presence of a biofilm in a standardized and reproducible manner, acceptable to regulatory agencies?
3.10 How can we educate society and policy makers about the origin, impact and importance of biofilms?
3.11 How can we increase impact of the term ‘biofilm' and raise awareness about the concept and its significance with industry, regulators and the public?
3.12 Is the lack of recognition of biofilms within healthcare and wider industrial regulatory systems blocking innovation and translation? If so, does this reduce the incentive to innovate? What can be done about this?
3.13 How can we accurately quantify biofilm biomass to assess biofilm removal/cleaning?
3.14 What new tools and methods/techniques can be developed to track the dynamics of biofilm development from initial adhesion through biofilm maturation?
3.15 During biofilm formation, various gradients will create specific microenvironments, local adaptations, and differentiation. Can we build models that predict structure and responses of biofilms based on their metabolic fluxes? Can we use metabolism and metabolic modeling to compare biofilms formed in different conditions?

### 3.4 Polymicrobial, interactions, ecology, microbiome, phage

In the past decades we have witnessed tremendous progress in advancing and refining the paradigms of biofilm development and the mechanisms that govern biofilm formation, structure, activity and dynamics. A major outstanding challenge in which the field is now engaged in is to move these discoveries beyond single species communities and develop them into principles and controls for multispecies communities, characteristic of real-world settings. Understanding the dynamics, metabolism and controls for complex polymicrobial communities will transform our ability to design and integrate novel and precision strategies to kill, remove, detect, control, engineer and manage microbial systems and microbiomes across multiple environments and end-uses. This will also require understanding of microbial interactions with the virome, e.g. it is now clear that bacteriophages impact greatly on biofilm dynamics, structure and virulence [61,62]. There is huge interest in the potential for bacteria-bacteriophage interactions to provide precision targeting and control of the composition of microbial communities, biotechnology applications, and in combatting AMR [63,64]. In the context of the host interactions with biofilms and microbiomes, key challenges include understanding and benchmarking ‘healthy’ microbial communities and how these transition to a diseased state [65,66]. An additional challenge in this area of research is that biobanks and standardized collections of biofilm samples relevant to health and industrial applications are lacking. They are required not only for fundamental research on the biology of biofilms but also for the relevant testing and validation of novel interventions, and well-characterised biobanks of polymicrobial communities that retain their physicochemical and biological properties upon storage would be hugely beneficial [67].

The identified priority questions in this category are:

4.1 Should we focus more biofilm research on polymicrobial biofilm models to complement studies on pure cultures?
4.2 Can we develop polymicrobial biofilm models that can be used widely by the community?
4.3 What are the physical, chemical, and biological mechanisms that underpin species interactions and support diversity in biofilms?
4.4 How can we increase our understanding of heterogeneity in structurally complex biofilms?
4.5 How does biofilm structure mediate its function, especially the emergent properties that arise from structure?
4.6 What is the role of bacteriophages and other viruses in the formation and development of biofilms?
4.7 Can we exploit bacteriophages to control or engineer biofilm communities?
4.8 What are the molecular mechanisms used by lytic phages to hijack biofilm dormant cells?
4.9 Complex, mature biofilms remain difficult to control, even with aggressive biocides. Are there new and innovative ways to disrupt and remove established biofilms?
4.10 Could pre-formed biofilms offer advantages in probiotic approaches to ‘eco-engineering'?
4.11 How do we detect/characterize when a biofilm transitions from a healthy to unhealthy, pathogenic or dysbiotic state?
4.12 What is the role of bacterial produced extracellular vesicles in biofilm formation and persistence?
4.13 How do (micro-)aggregates form? Does this process fundamentally differ from the formation of surface-attached biofilms?

### 3.5 Clinical focus, chronic infection, detection, diagnostics

Guidelines for improved diagnosis of biofilm-associated infections have been published [34,35] but the need for rapid and accurate detection of the presence of a biofilm and identification of the organisms in the biofilm, in various settings, remains high. We currently lack reliable biomarkers for diagnosis and while some risk factors for chronic biofilm-related infection associated with implanted medical devices have been identified (and appear to be mainly related to a compromised innate immune response) [68], we overall lack the capability to predict the likelihood of biofilm-associated infections occurring in a specific patient or in patient groups. Microscopic methods, including methods based on fluorescent *in situ* hybridization and/or confocal laser scanning microscopy, as well as newer approaches like scanning probe microscopies, spatially resolved spectroscopies and smart sensors are very valuable for confirming presence of biofilms [[69], [70], [71]], although they currently remain poorly-suited for use in routine diagnostic settings. Alternative methods for detecting biofilms, including biosensors [[72], [73], [74], [75]] and microcalorimetry [76] are promising, but more validation is required prior to clinical implementation. Continuing innovations in (metagenomic) sequencing as well as in other omics-approaches have translated to clinical microbiology (e.g. in the diagnosis of prosthetic joint infections [77,78]) and they may provide additional ways to provide in-depth insights into the composition of the biofilm. An older but recurring question pertains to the use of reference isolates (‘lab strains’), rather than recent (clinical and/or environmental) isolates [79,80]. While the use of reference isolates in theory improves portability of data (i.e. allows comparing data obtained in multiple labs) there may be extensive diversity between ‘the same’ reference strain in different labs (as for example shown for *Pseudomonas aeruginosa* PAO1 [81]) and reference strains may not reflect the diversity found among recent clinical isolates (as for example shown for *Acinetobacter baumannii* [82]).

The identified priority questions in this category are:

5.1 How can we develop rapid, non-invasive, *in situ*, point-of-use detection methods, applicable across a range of sectors?
5.2 How can we carry out direct *in situ* detection and characterization of biofilms in patient samples?
5.3 Are there specific biomarkers for biofilms?
5.4 Can we identify biomarkers that allow accurate diagnosis and monitoring of biofilm infections (e.g. to determine when it is safe to stop antibiotic treatment)?
5.5 Can we predict which patients are more likely to develop implant-associated infections?
5.6 The majority of biofilm data is generated using lab strains. How do we translate this data if it is not carried out using clinically or environmentally relevant strains?
5.7 Can we create improved methods for detection of biofilms and monitoring of systems in inorganic/industrial settings (e.g. deployable, accurate, sensitive biofilm/corrosion sensors)?
5.8 Can we detect the presence of a biofilm in real time?
5.9 How can we improve models and methods for characterization, visualization and detection of biofilms: relevant (real world context), standardized and accessible?
5.10 Can structures common to all biofilms be identified and used for detection?
5.11 How can we identify the main microorganisms present in biofilms in chronically infected sites in a reasonably short period of time without the use of expensive and elaborate protocols?
5.12 There are many biofilm detection techniques based on physical, chemical, microscopical, and biological aspects of the biofilm formation; how should we combine this myriad of techniques in order to create standard methods?
5.13 What revisions to standard tests and diagnostic methods are needed to distinguish the biofilm phenotype from general bacterial presence, and in which sector is this most important?

### 3.6 Matrix, lipids, capsule, metabolism, development, physiology, ecology, evolution, environment, microbiome, community engineering

One of the key properties of biofilm communities is that they are embedded in a (at least partially) self-produced matrix consisting of a wide range of extracellular polymeric substances (EPS) [3,83]. A wide range of functions have been attributed to the biofilm matrix, and while there are few studies on the *in vivo* role of the biofilm matrix, evidence is accumulating that it is for example important for *in vivo* tolerance to antimicrobial treatment [84,85]. The species composition of a biofilm influences the matrix composition [86] but few tools are currently available to study this in physiologically relevant conditions [87]. Microbial biofilms of course do not exist in isolation, and *in vivo* there will be interactions with many other (micro)organisms [88] but much remains to be learned about how these interactions co-determine structure and function of biofilms. A specific set of interactions are these between microbial biofilms on the one hand, and humans, animals and plants on the other. These interactions play a crucial role in health and disease, and form the basis for community engineering, yet remain to be fully understood [89,90]. Compared to their planktonic counterparts, biofilm-associated microorganisms often have different metabolic properties (partly related to biofilm heterogeneity and the microenvironment), that can profoundly influence their properties [29,[91], [92], [93]], although the exact contributions of many of these metabolic changes to overall biofilm biology remain elusive [94]. Mutation rates and frequency of horizontal gene transfer are typically higher in biofilms than in planktonic populations, but most data in this context are derived from *in vitro* studies and an in depth understanding of how evolutionary processes shape biofilm biology is lacking [30]. Finally, microbial biofilms are not the only three-dimensional multicellular structures that form in nature, and similarities between biofilms and solid tumors have been noticed but not explored in detail [95,96].

The identified priority questions in this category are:

6.1 How can we understand the mechanism of the interaction of antibiotics with biofilms?
6.2 Is there any relationship between the biofilm matrix components and the development of non-infectious diseases such as cancer, neurological disorders or inflammatory diseases?
6.3 How does matrix alteration contribute to resilience of biofilms?
6.4 How do we develop better models to study host-microbiome (biofilm) interactions, also beyond infection models?
6.5 Are there commonalities in the relationships between microbial communities, their structure, function and their environment that would allow for global predictions of complex biofilm behaviors?
6.6 Can we distinguish structure/conformation/function of macromolecules (eRNA/eDNA/eProteins, etc.) that are intra-vs extra-cellular (part of matrix)?
6.7 How do natural multi-species biofilm members alter the matrix produced by the whole community?
6.8 How do we track spatial/temporal organization of matrix components vs microorganisms in the biofilm?
6.9 What can we learn from the study of three-dimensional solid cancers?
6.10 Which components or characteristics of biofilms are most widely conserved between biofilms from different systems?
6.11 What is the lipidome of a biofilm? How it is different from the lipidome of a planktonic microorganism? What are the dynamics of the biofilm lipidome during a biofilm cycle?
6.12 What is the rate of genetic evolution in different types of biofilm?
6.13 Why is biofilm diversity (or fitness) in the natural environment so different from the lab?
6.14 In mixed-species biofilms, how do the extracellular polymeric substances (EPS) produced by each member contribute to the biofilm matrix? Are different types of EPS (in)compatible to build the biofilm matrix?

## 4 Concluding remarks

The success and relevance of priority questions exercises like the one described here depends on two crucial factors. First of all, it needs to be based on input from a diverse set of domain experts. While we managed to achieve overall balance in terms of gender, geographical distribution (although there is an underrepresentation of participants from the Global South) and subdisciplines covered, it is clear we did not manage to fully capture the input of people active in the field of biofilms outside academia, and this is an important point of attention for the future. Secondly, the selection and grouping of questions into meaningful themes should be as unbiased as possible and lead to a manageable number of questions grouped into broad clusters or themes that are internally coherent. Using a hybrid approach of manual data curation by experts and computer-driven groupings by experts, we believe we accomplished this goal.

The 78 questions identified in this priority questions exercise highlight the areas in which there are opportunities to go beyond the state-of-the-art. They also highlight the barriers in translation and technology development that need to be overcome in order to deliver benefit to society. We hope these questions will stimulate (interdisciplinary) discussions among researchers in academia and industry, as well as policy makers.

Finally, not all topics worthy of the label ‘priority’ may have been addressed in the current list. In addition, priorities will change over time with advances in science and changes to policies. While we believe the priority questions identified in this exercise will remain valid in the near future, it is clear that this is not a static document and that biofilm research and policy priorities will need to be readdressed on a frequent basis.

## CRediT authorship contribution statement

**Tom Coenye:** Writing – original draft, Methodology, Investigation, Formal analysis, Data curation, Conceptualization. **Merja Ahonen:** Writing – review & editing, Methodology, Data curation, Conceptualization. **Skip Anderson:** Investigation, Data curation, Conceptualization. **Miguel Cámara:** Writing – review & editing, Data curation, Conceptualization. **Parvathi Chundi:** Methodology, Formal analysis, Data curation, Conceptualization. **Matthew Fields:** Methodology, Formal analysis, Data curation, Conceptualization. **Ines Foidl:** Writing – review & editing, Project administration, Methodology, Data curation, Conceptualization. **Etienne Z. Gnimpieba:** Methodology, Investigation, Formal analysis, Data curation. **Kristen Griffin:** Project administration, Methodology, Data curation, Conceptualization. **Jamie Hinks:** Data curation, Conceptualization. **Anup R. Loka:** Methodology, Formal analysis, Data curation, Conceptualization. **Carol Lushbough:** Methodology, Investigation, Formal analysis, Data curation. **Cait MacPhee:** Writing – review & editing, Data curation, Conceptualization. **Natasha Nater:** Project administration, Methodology, Conceptualization. **Rasmita Raval:** Writing – review & editing, Data curation, Conceptualization. **Jo Slater-Jefferies:** Writing – review & editing, Data curation, Conceptualization. **Pauline Teo:** Project administration, Data curation, Conceptualization. **Sandra Wilks:** Writing – review & editing, Data curation, Conceptualization. **Maria Yung:** Data curation, Conceptualization. **Jeremy S. Webb:** Writing – review & editing, Writing – original draft, Methodology, Formal analysis, Data curation, Conceptualization.

## Declaration of competing interest

The authors declare the following financial interests/personal relationships which may be considered as potential competing interests:

Given his role as Co-Editor in Chief, TOM COENYE had no involvement in the peer review of this article and has no access to information regarding its peer review. Full responsibility for the editorial process for this article was delegated to BIRTHE KJELLERUP. If there are other authors, they declare that they have no known competing financial interests or personal relationships that could have appeared to influence the work reported in this paper.

## Data Availability

No data was used for the research described in the article.

## Appendix A Supplementary data

Supplementary Table S1 Excel file with all questions. Supplementary Table S1

Supplementary Table S2 Biofilm Priority Questions Exercise Participants. Supplementary Table S2

Supplementary Fig. S1 Empty submission form. Supplementary Fig. S1

Supplementary Fig. S2 Some examples of tweets posted to invite submissions. Supplementary Fig. S2

Supplementary Fig. S3 Example of an invitation mail. Supplementary Fig. S3
