# Supplementary material for: Global challenges and microbial biofilms: Identification of priority questions in biofilm research, innovation and policy
Source: Biofilm. 2024 Jul 4;8:100210. doi: 10.1016/j.bioflm.2024.100210 (PMC11364012; doi:10.1016/j.bioflm.2024.100210)
Supplement: Supplementary Table S2 — Biofilm Priority Questions Exercise Participants. [file mmc2.pdf]

Supplementary Table S2. Biofilm Priority Questions Exercise Participants and their affiliation (only participants that were not part of the core working group are listed)

Irina Afonina (Biomed Global, Singapore)
Claudio Angione (Teesside University, UK)
Haris Antypas (SCELSE, Singapore)
Valérie Baede (National Institute for Public Health and the Environment, The Netherlands)
Pascale B. Beauregard (Université de Sherbrooke, Canada)
Marilyn Bruno (Aequor Inc., US)
Anthony Buckley (University of Leeds, UK)
Mette Burmølle (University of Copenhagen, Denmark)
Yuming Cai (Ghent University, Belgium)
Ashwini Chauhan (University of Delhi South Campus, India)
Dan Cheng (National Technological University/SCELSE, Singapore)
Suhelen Egan (UNSW Sydney, Australia)
Bruno Haas (STERIS, Canada)
Suzie Hingley-Wilson (University of Surrey, UK)
Megan Kiedrowski (University of Alabama at Birmingham, US)
Iñigo Lasa (Navarrabiomed/Universidad Publica de Navarra, Spain)
Yongqiang Liu (University of Southampton, UK)
Robert J.C. McLean (Texas State University, US)
Luis F. Melo (University of Porto, Portugal)
Daniel Metcalf (ConvaTec, UK)
Rikke Louise Meyer (Aarhus University, Denmark)
Kasper Nørskov Kragh (Symcel, Denmark)
María Gabriela Paraje (Natural Sciences National University of Córdoba, Argentina)
Elinor Pulcini (Montana State University-Bozeman/CBE, US)
Fany Reffuveille (University of Reims Champagne Ardenne, France)
Constantinos Regas (University of Southampton, UK)
Paul Renick (Beckman Coulter Diagnostics, US)
Santiago Romero-Vargas Castrillon (University of Edinburgh, UK)
Tony Rook (Sherwin-Williams Company, US)
Mariana Spesia (Consejo Nacional de de Investigaciones Científicas y Técnicas/Universidad Nacional de Rio Cuarto, Argentina)
Paul Stoodley (The Ohio State University, US)
Chaimaa Tarzi (Teesside University, UK)
Lara Thieme (Jena University Hospital, Germany)
Alex Valm (SUNY Albany, US)
Yissue Woo (SCELSE, Singapore)
Thomas K. Wood (Pennsylvania State University, US)
Yvonne Hii Yiik Siang (SCELSE, Singapore)
Arsalan Zaidi (National Institute for Biotechnology and Genetic Engineering, Pakistan)
