# Supplementary material for: Global challenges and microbial biofilms: Identification of priority questions in biofilm research, innovation and policy
Source: Biofilm. 2024 Jul 4;8:100210. doi: 10.1016/j.bioflm.2024.100210 (PMC11364012; doi:10.1016/j.bioflm.2024.100210)
Supplement: Supplementary Fig. S1 — Empty submission form. [file mmc3.pdf]

Biofilms Priority Questions Exercise

An initiative launched by the Center for Biofilm Engineering (CBE), COST AMiCI, the ESCMID Study Group for Biofilms (ESGB), the National Biofilms Innovation Centre (NBIC) and the Singapore Centre for Environmental Life Sciences Engineering (SCELSE) in order to crowd-source an international and community-wide synthesis of key questions and priority research or innovation areas for the biofilm field. More information here: https://www.biofilms.ac.uk/biofilm-priority-questions

* Required

1. Provide your priority question *

   Questions should be less than 100 words and should not have already been answered within the scientific literature.

2. Please provide some key words for this question *

   The key words should be separated by a semicolon.

3. Would you like to submit more questions? *

   ◯ Yes

   ◯ No

4. Provide another priority question *

   Questions should be less than 100 words and should not have already been answered within the scientific literature.

5. Please provide key words *

   The key words should be separated by a semicolon.

6. Would you like to submit more questions? *

- ◯ Yes
- ◯ No

7. Provide another priority question *

Questions should be less than 100 words and should not have already been answered within the scientific literature. If you'd like to submit more questions, please complete another form.

8. Please provide key words *

The key words should be separated by a semicolon.

9. Which field do you consider yourself to be part of? *

Check all that apply.

- ☐ Academia
- ☐ Funding Organisation
- ☐ Government Agency
- ☐ Industry
- ☐ Policy-making
- ☐ Trade Organisation
- ☐ Other

10. Which discipline or sector do you consider yourself to be in? *

Check all that apply.

- [ ] Biological Sciences
- [ ] Chemistry
- [ ] Energy
- [ ] Engineering
- [ ] Environmental Sciences
- [ ] Food
- [ ] Home and Personal Care
- [ ] Marine
- [ ] Medicine and Health
- [ ] Pharmaceuticals
- [ ] Physics
- [ ] Water
- [ ] Other

11. Which country are you based in? *

- ◯ Afghanistan
- ◯ Albania
- ◯ Algeria
- ◯ Andorra
- ◯ Angola
- ◯ Antigua and Barbuda
- ◯ Argentina
- ◯ Armenia
- ◯ Australia
- ◯ Austria
- ◯ Azerbaijan
- ◯ Bahamas
- ◯ Bahrain
- ◯ Bangladesh
- ◯ Barbados
- ◯ Belarus
- ◯ Belgium
- ◯ Belize
- ◯ Benin
- ◯ Bhutan
- ◯ Bolivia
- ◯ Bosnia and Herzegovina
- ◯ Botswana
- ◯ Brazil
- ◯ Brunei Darussalam
- ◯ Bulgaria
- ◯ Burkina Faso
- ◯ Burundi
- ◯ Cabo Verde
- ◯ Cambodia

- Cameroon
- Canada
- Central African Republic
- Chad
- Chile
- China
- Colombia
- Comoros
- Congo
- Costa Rica
- Côte D'Ivoire
- Croatia
- Cuba
- Cyprus
- Czech Republic
- Democratic People's Republic of Korea
- Democratic Republic of the Congo
- Denmark
- Djibouti
- Dominica
- Dominican Republic
- Ecuador
- Egypt
- El Salvador
- Equatorial Guinea
- Eritrea
- Estonia
- Eswatini
- Ethiopia
- Fiji
- Finland
- France

- Gabon
- Gambia
- Georgia
- Germany
- Ghana
- Greece
- Grenada
- Guatemala
- Guinea
- Guinea Bissau
- Guyana
- Haiti
- Honduras
- Hungary
- Iceland
- India
- Indonesia
- Iran
- Iraq
- Ireland
- Israel
- Italy
- Jamaica
- Japan
- Jordan
- Kazakhstan
- Kenya
- Kiribati
- Kuwait
- Kyrgyzstan
- Lao People's Democratic Republic

- Latvia
- Lebanon
- Lesotho
- Liberia
- Libya
- Liechtenstein
- Lithuania
- Luxembourg
- Madagascar
- Malawi
- Malaysia
- Maldives
- Mali
- Malta
- Marshall Islands
- Mauritania
- Mauritius
- Mexico
- Micronesia
- Monaco
- Mongolia
- Montenegro
- Morocco
- Mozambique
- Myanmar
- Namibia
- Nauru
- Nepal
- Netherlands
- New Zealand
- Nicaragua
- [illegible]

- Niger
- Nigeria
- North Macedonia
- Norway
- Oman
- Pakistan
- Palau
- Panama
- Papua New Guinea
- Paraguay
- Peru
- Philippines
- Poland
- Portugal
- Qatar
- Republic of Korea
- Republic of Moldova
- Romania
- Russian Federation
- Rwanda
- Saint Kitts and Nevis
- Saint Lucia
- Saint Vincent and the Grenadines
- Samoa
- San Marino
- Sao Tome and Principe
- Saudi Arabia
- Senegal
- Serbia
- Seychelles
- Sierra Leone
- Singapore

- Slovakia
- Slovenia
- Solomon Islands
- Somalia
- South Africa
- South Sudan
- Spain
- Sri Lanka
- Sudan
- Suriname
- Sweden
- Switzerland
- Syrian Arab Republic
- Tajikistan
- Thailand
- Timor-Leste
- Togo
- Tonga
- Trinidad and Tobago
- Tunisia
- Turkey
- Turkmenistan
- Tuvalu
- Uganda
- Ukraine
- United Arab Emirates
- United Kingdom of Great Britain and Northern Ireland
- United Republic of Tanzania
- United States of America
- Uruguay
- Uzbekistan

- Vanuatu
- Venezuela
- Viet Nam
- Yemen
- Zambia
- Zimbabwe
- Other
