# Supplementary material for: Global challenges and microbial biofilms: Identification of priority questions in biofilm research, innovation and policy
Source: Biofilm. 2024 Jul 4;8:100210. doi: 10.1016/j.bioflm.2024.100210 (PMC11364012; doi:10.1016/j.bioflm.2024.100210)
Supplement: Supplementary Fig. S2 — Some examples of tweets posted to invite submissions. [file mmc4.pdf]

ESGB
@esgb_escmid

Support our call to identify issues important to the microbial #biofilm field! We're looking for questions that will impact scientists, policy makers & funders. Deadline: 31 March. Visit biofilms.ac.uk/biofilm-priori ... to participate. #BiofilmPQs @nbic @Center4Biofilm @SCELSE_SG @ESCMID

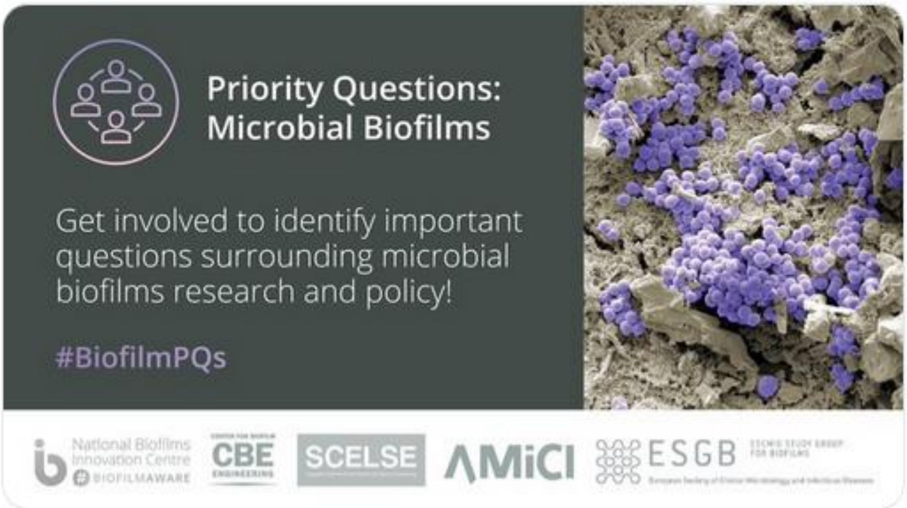

8:30 AM · Mar 16, 2021

National Biofilms Innovation Centre (N... @ukbiofi... · Mar 30, 2021

🔔 Don't forget to submit your key questions by 31 March!

🧑‍🔬Help us identify those that, if answered, will make a considerable impact to the microbial #biofilm field!🔬

🔜 biofilms.ac.uk/PQs

#BiofilmPQs @esgb_escmid @Center4Biofilm @SCELSE_SG @AMiCIConsortium #biofilms

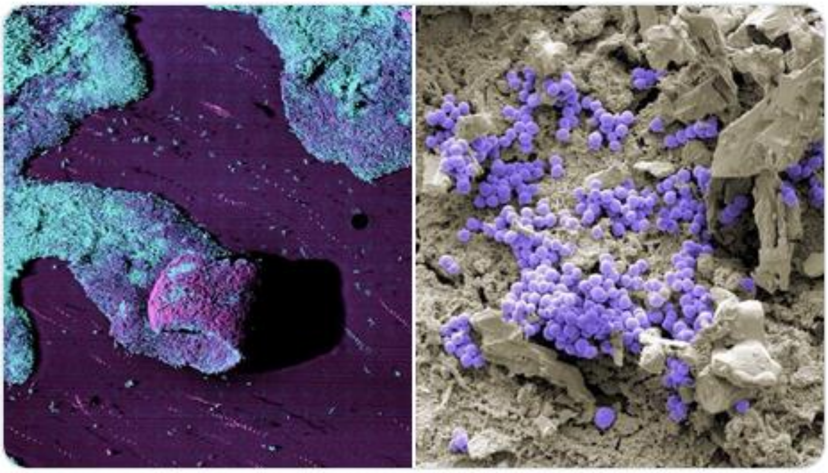

Applied Microbiology International and 9 others

The Center for Biofilm Engineering @BiofilmFirst · Mar 8, 2021 ···

Support our international call to identify emerging issues in science, innovation & policy for the microbial #biofilm field! We're looking for questions that, if answered, will make a huge impact in influencing policy makers & funders bit.ly/cbe-biofilm-pqs **#BiofilmPQs**

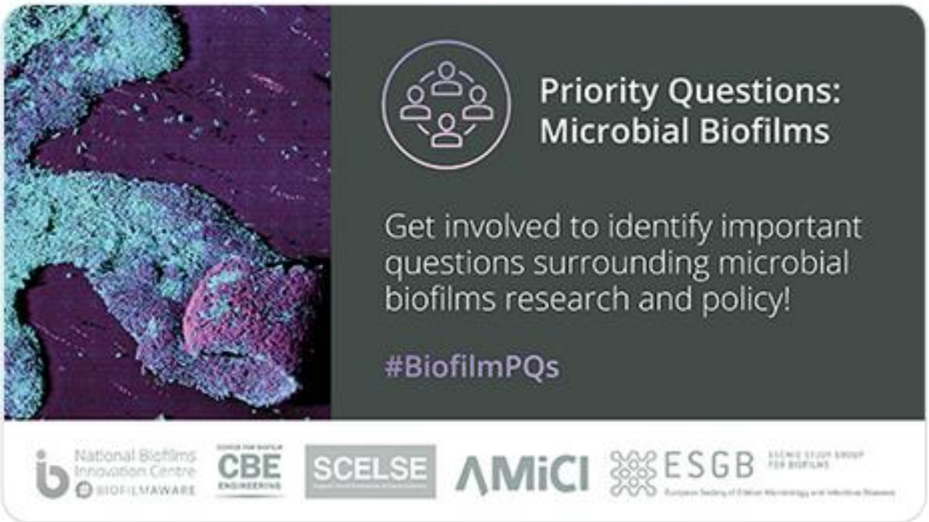

SCELSE @SCELSE_SG · Feb 18, 2021 ···

Support our international call to identify emerging issues in #science, innovation, policy on microbial #biofilms! Seeking questions that will make a huge impact in influencing policy makers & funders:

👉 biofilms.ac.uk/biofilm-priori...

#BiofilmPQs #BiofilmAware #BiofilmQuestions

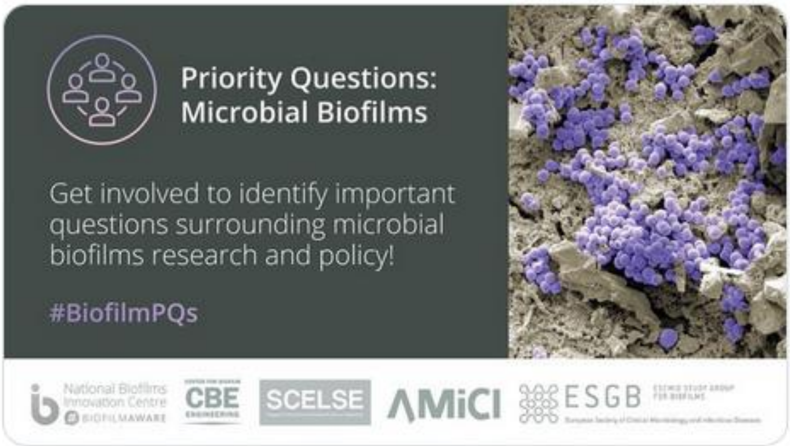

National Biofilms Innovation Centre (NBIC) and 4 others
