# Supplementary material for: Global challenges and microbial biofilms: Identification of priority questions in biofilm research, innovation and policy
Source: Biofilm. 2024 Jul 4;8:100210. doi: 10.1016/j.bioflm.2024.100210 (PMC11364012; doi:10.1016/j.bioflm.2024.100210)

To date, there has not been an international and community-wide synthesis of key questions and priority research or innovation areas for the biofilm field. Such exercises can play a critical role in bridging the gap between the data generated by researchers, and the information needed by policymakers to make funding or regulatory decisions.

[...]the Center for Biofilm Engineering (CBE), Singapore Centre for Environmental Life Sciences Engineering (SCELSE), COST AMiCI Consortium, the ESCMID Study Group for Biofilms (ESGB), and the National Biofilms Innovation Centre (NBIC) [...] are calling on the international biofilms community to help us identify important questions that, if answered, will make a considerable impact onthe fundamentals of the field of microbial biofilms, to innovation in approaches to prevent, detect, manage and engineer biofilms, or which would be expected to have an impact in influencing policy makers andfunders. We are calling for questions that are unanswered, could be answered (including through high-risk and blue-skies research), and that could be tackled by a research programme.

Goals

- To stimulate discussion amongst the biofilm community and identify areas of research and innovation that would have a substantial scientific and societal impact.
- To encourage researchers to think beyond the limits of their own sphere of research or discipline and consider the most important basic or applied research that could possibly be carried out.
- To illustrate the most impactful and beneficial research in the field and its overall importance to funding agencies, policy makers, regulators and the wider public.

Our definition of microbial biofilms is as inclusive as possible and includes communities of bacteria that may be surface or interface-associated or suspended as aggregates, comprising single-species or polymicrobial consortia, and relevant to any fundamental or applied context in which biofilms are studied.
To participate, please submit your questions via our short online form by 31 March 2021. Once you have entered your question(s) anonymously into the form you will also have an option to receive further updates on the Biofilms Priority Questions initiative.

The process

All questions are submitted anonymously. Once our call for questions closes in March, all submissions will be pre-screened for duplication before being grouped into themes. We will then hold thematic focus groups, which will include scientists from research and industry, policymakers and other stakeholders, to organise subsets of related questions and finalise our set of priority questions. These will form the basis for a position paper, which will be published as a resource for the field. This is expected to help set the agenda for future research in the field of microbial biofilms as well as have impact in areas of policy and outreach. After submitting your question, if you are interested in participating further in the process you will have the opportunity to indicate this on the question form

which will only take a few minutes to fill in. Questions should be less than 100 words and should not have already been answered within the scientific literature.

Support
If you are able to support the promotion this initiative, you can find a number of digital assets available on NBIC's #BiofilmAware hub to share with your scientific community. Please feel free to share this with as many biofilm researchers (in- and outside your institution) as possible.

Background
In May 2019, 29 scientists with expertise in various subdisciplines of biofilm research met in the US at an event designated as the '2019 Biofilm Bash'. Click here to view the meeting report. The goal of the meeting was first to identify knowledge gaps, and then to come up with ways of how the biofilm community can fill these gaps. Among the outcomes of this meeting, it was identified that increased interdisciplinary and international networking would be beneficial and that the larger centres can and should play a role in catalysing these interactions, which would bring additional value (e.g., in leveraging international funding initiatives). A joint meeting of biofilm centres was held in Arlington, USA, February 2020, and the proposal is for the core international partners, NBIC, CBE, SCELSE, AMiCI and ESGB to work together with the community to coordinate a priority question exercise.

If you are interested in hearing more about taking part in the Biofilms Priority Questions initiative process please get in touch via priorityquestions@biofilms.ac.uk.

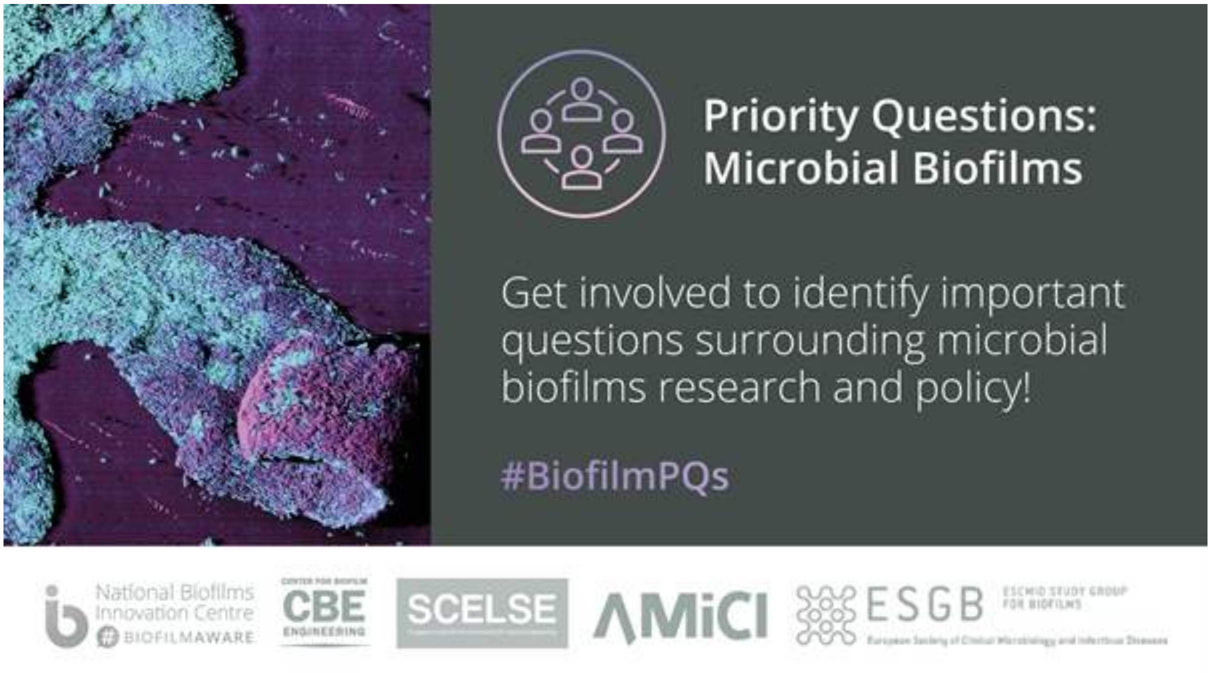

Supplement: Supplementary Fig. S3 — Example of an invitation mail. [file mmc5.pdf]
